# A qualitative interview study on colorectal cancer screening in China

**DOI:** 10.3389/fmed.2024.1232134

**Published:** 2024-01-31

**Authors:** Weimiao Wu, Songsong Tan, Junjie Huang, Yingyao Chen, Martin C. S. Wong, Wanghong Xu

**Affiliations:** ^1^Global Health Institute, Fudan University School of Public Health, Shanghai, China; ^2^The Jockey Club School of Public Health and Primary Care, Faculty of Medicine, Chinese University of Hong Kong, Shatin, Hong Kong SAR, China

**Keywords:** colorectal cancer, screening, initial tests, qualitative interview, grounded theory

## Abstract

**Background:**

The effectiveness of triage screening for colorectal cancer (CRC) is not fully achieved in Chinese populations, mainly due to low compliance to colonoscopy follow-up. This study aimed to collect viewpoints of experts in China on ongoing screening programs and emerging screening tests for CRC, which may help to improve effectiveness of CRC screening in the country.

**Methods:**

We conducted 15 semi-structured interviews with experts involving CRC screening in China during October to November of 2020. Interview topics included personal characteristics, work context, opinions on ongoing screening programs, challenges and opportunities in optimization of screening strategies, and prospects for CRC screening in near future. To analyze the data, we used a generic qualitative research approach inspired by grounded theory, including open, axial, and selective coding.

**Results:**

This analysis revealed a total of 83 initial categories, 37 subcategories and 10 main categories, which included 4 core categories of current modality for CRC screening, factors influencing screening effectiveness, optimization of CRC screening modality, and prospects for development of CRC screening. The results provide insight into the factors underlying the challenges of the ongoing CRC screening programs in China: the most important concern is the low compliance to colonoscopy, followed by the low specificity of the currently-used initial tests. The experts proposed to use quantitative instead of qualitative fecal immunochemical test (FIT), and optimize risk assessment tools to improve specificity of initial tests. Regarding the emerging screening tests, 9 of 15 experts did not think that the novel techniques are good enough to replace the current tests, but can be used complementarily in opportunistic screening for CRC.

**Conclusion:**

The viewpoints of Chinese experts suggested that use quantitative FIT or optimize risk assessment tools may help to identify high-risk individuals of CRC more accurately, improve adherence to colonoscopy, and thus fully achieve the effectiveness of screening.

## 1 Introduction

The incidence and mortality of colorectal cancer (CRC) have been greatly reduced by large-scale screening for lesions in colon and rectum among average-risk populations (1). Many countries and areas have provided organized CRC screening for middle-aged and elderly populations as local or national public health service programs (2). The triage screening strategy, mostly colonoscopy referral for individuals with positive fecal immunochemical test (FIT) results, was widely adopted in the programs (3, 4). However, FIT is not sensitive to non-bleeding lesions. Therefore, multiple risk scoring systems have been developed and used combinatorically with FIT to identify high-risk individuals for subsequent colonoscopy, particularly in Asia-Pacific countries with a low incidence of the cancer (5).

In China, the questionnaire-based risk assessment (RA) and two-sample qualitative FIT has been parallel used to detect CRC since 2005 (6). The parallel tests have been proved cost-effective in Chinese populations (7), and were recommended as initial tests for CRC screening programs in China. So far, however, the public health service programs were provided only in Shanghai (8), Guangzhou (9), Tianjin (10), Hangzhou (11), and other urban areas, including the population-based Cancer Screening Program in Urban China (CanSPUC) program that covered 22 cities in 16 provinces and used a risk scoring system incorporating previous fecal occult blood test results (12). The utility of the RA tools, however, has been consistently observed to result in suboptimal adherence to colonoscopy, which greatly jeopardized the efficiency of CRC screening (11–14). In our previous studies, we found that the colonoscopy adherence was less than 40%, and positively related with the specificity of initial screening tests (14, 15).

With the development of biotechnology, a number of novel tests have been developed in recent years, in which colon capsule endoscopy, computed tomographic colonography, and molecular biomarkers in stool or blood at DNA, RNA and protein levels are promising in CRC screening (16, 17). Of the novel biomarkers, multi-target stool DNA test (mt-sDNA) and methylated *SEPT9* DNA plasma assay (m*SEPT9*) have been recommended as complementary tests for FIT in the 2016 USPSTF guideline (18). These emerging technologies provide multiple choices of screening tests, which may break the bottlenecks of the current screening modalities and promote optimization and diversification of screening strategies.

To better understand the advantages and disadvantages of the currently-used screening tests for CRC in China, and the potential applications of the novel tests in large-scale CRC screening practices, we conducted a qualitative study based on grounded theory. The grounded theory focuses on revealing the process of a phenomenon and the diverse perspectives regarding the phenomenon, thereby developing an explanatory theory for this phenomenon (19). The theory emphasizes the theoretical sampling, constant comparison of data, and theoretical saturation. The simultaneous data collection and analysis allow theoretical sampling of interviewees who can provide information to develop a theory and finally reach theoretical saturation (20).

The grounded theory provides an ideal qualitative methodological framework to explore viewpoints of experts on the situation of CRC screening and the utility of novel screening tests in China, which may help to optimize and update the screening guidelines, facilitate identification of high-risk individuals for colonoscopy, and improve efficiency of CRC screening programs in China.

## 2 Methods

### 2.1 Study design

In this qualitative study utilizing an exploring research technology of grounded theory (19), a semi-structured open-ended individual interview was conducted in China from October to November of 2020. The study was reported according to the Consolidated Criteria for Reporting Qualitative Research (COREQ) reporting guideline (21).

This study was approved by the Ethics Committee of Fudan University School of Public Health (IRB00002408 & FWA00002399) (Registration number: 2019-TYSQ-03-29). Prior to the commencement of each interview, each participant was informed of the purpose of the study and the voluntary, anonymous, and confidential nature of the interview using an information sheet. All participants were also informed of that the long interview period would cause minimal discomfort, and their withdrawal from the study would be permitted without any adverse outcomes. The participants were required to sign an informed consent form before taking part. The authors declare that the study was carried out in accordance with the Declaration of Helsinki for research involving human subjects (22).

### 2.2 Study participants

To ensure adequate representativeness and diversity of the viewpoints, the study participants were selected from different health sections of government, research institutes, centers for disease prevention and control (CDC), hospitals, and community healthcare centers in China using a purposive sampling method. Their profession, position, experience, and knowledge in CRC screening were also considered to ensure the full coverage of the service delivery (i.e., policy-making, administration, supervision, organization, implementation, follow-up, diagnosis and treatment, and evaluation). The potential experts were invited to participate the interview by phone-call or sending an email, and then participants were scheduled for an interview at a convenient time. A total of 15 participants were interviewed. The participants’ characteristics are presented in Table 1.

**Table 1:** Characteristics of the 15 experts interviewed.

ID	Qualitative research technique	Gender	Age group (years)	Level of education	Occupation	Professional title	Roles in CRC screening^*^	Experience in CRC screening (years)
1	Online interview	Male	55–59	Undergraduate or below	Primary care provider	Senior	a, c, d	23
2	Online interview	Male	40–44	Undergraduate or below	Gastroenterologist	Intermediate	a, c, d, e	5
3	Face-to-face interview	Male	50–54	Master	Gastroenterologist	Vice-senior	b, d, e	15
4	Face-to-face interview	Male	40–44	MD, PhD	Gastroenterologist	Vice-senior	c, e	15
5	Online interview	Male	40–44	Master	Government health official	Intermediate	c	8
6	Online interview	Female	40–44	MD, PhD	Scientific researcher	Vice-senior	a	8
7	Face-to-face interview	Female	40–44	MD, PhD	CDC staff member	Senior	a, b, c	10
8	Online interview	Female	<35	Undergraduate or below	Primary care provider	Primary	d	3
9	Face-to-face interview	Female	<35	Undergraduate or below	Primary care provider	Primary	b	5
10	Online interview	Female	40–44	Master	CDC staff member	Vice-senior	c, d	5
11	Face-to-face interview	Female	45–49	Master	CDC staff member	Senior	a, c, d	10
12	Online interview	Female	40–44	MD, PhD	Scientific researcher	Vice-senior	a	10
13	Online interview	Male	35–39	MD, PhD	Gastroenterologist	Vice-senior	a, d, e	10
14	Online interview	Male	<35	MD, PhD	Scientific researcher	Vice-senior	a, b, c, d	7
15	Online interview	Male	45–49	MD, PhD	Scientific researcher	Senior	a, b, c	5

CRC: colorectal cancer; CDC: centers for disease prevention and control. ^*^ a: scientific research activities; b: development of guidelines or recommendations; c: organization or supervision; d: implementation of screening; e: diagnosis or treatment for colorectal lesions.

### 2.3 Data collection

In-depth interviews were conducted using an interview outline conceptualized and developed based on the Consolidated Framework for Implementation Research (CFIR) (23), which has been widely used in health service research (24). The five major domains of CFIR-intervention characteristics, outer setting, inner setting, characteristics of individuals, and implementation process—have been used to practically guide the evaluation of barriers and facilitators of interventions (23), and therefore may be suitable for the assessment of the CRC screening.

**Figure 1:**
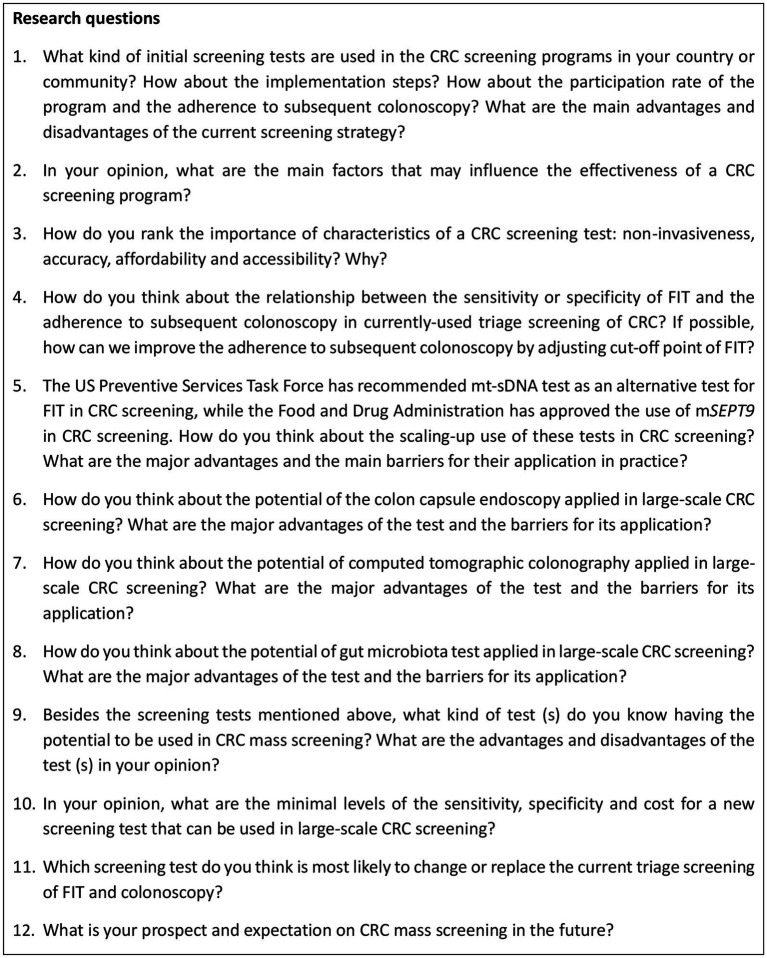
Interview guide to explore viewpoints of experts on CRC screening. CRC: colorectal cancer; FIT: fecal immunochemical test; m*SEPT9*: methylated *SEPT* DNA plasma assay; mt-sDNA: multi-target stool DNA test.

Based on systematic iterations of scientific literature reviews and careful selection and rephrasing of the items, we developed an interview outline including 12 most suitable probing questions concerning the current CRC screening protocol, barriers and facilitators for screening effectiveness, existing problems and potential optimization of screening protocol, and future prospects of CRC screening programs (Figure 1). Further discussion was performed along with the probing questions. We also collected reported sensitivity, specificity and price of currently-used initial screening tests through comprehensive literature reviews and consults with related experts. The information summarized in Table 2 was provided to the participants as a reference at the interview, which was supplemented at the same time by the experts interviewed. The protocol of the interview was pilot-tested with one expert (ID11) to determine the clarity, reliability, and convergent validity of the questions. As the pilot interview just indicated minor adjustment of the item order and language expression, but did not reveal any need for major modifications to the interview schedule, the results of the pilot test were incorporated in this analysis.

**Table 2:** Previous reported sensitivity, specificity, and cost of each screening test for colorectal cancer.

Screening test	Sensitivity (95% CI)	Specificity (95% CI)	Cost for each test (USD)		
	Reported value (reference)	Reported value (reference)	US hospitals	CMS (reference)	China (reference)
Risk assessment	0.25 (0.20, 0.30) (7)	0.898 (0.896, 0.900) (7)	–	–	0.5 (25)
gFOBT	0.60 (0.35, 0.84) (26)	0.90 (0.84, 0.94) (26)	49	–	–
Qualitative FIT	0.93 (0.83, 0.97) (27)	0.91 (0.88, 0.92) (27)	109	22 (28)	1.3 (29)
Quantitative FIT	0.86 (0.68, 0.95) (27)	0.91 (0.87, 0.94) (27)	109	22 (28)	2.8–11.2
mt-sDNA	0.92 (0.83, 0.98) (30)	0.90 (0.89, 0.91) (30)	–	512 (28)	137.2–280.0
Gut microbiota test	0.71 (0.61, 0.79) (26)	0.76 (0.66, 0.84) (26)	901	–	70 (26)
m*SEPT9*	0.63 (0.58, 0.67) (31)	0.91 (0.90, 0.92) (31)	606	192 (28)	119–140
Computed tomographic colonography	0.95 (0.90, 0.98) (32)	0.98 (0.95, 0.99) (32)	–	287 (28)	112–140
Colon capsule endoscopy	0.90 (0.79, 0.96) (33)	0.66 (0.57, 0.74) (33)	2,600	950 (34)	630–1,120
Colonoscopy without polypectomy	0.95 (0.92, 0.99) (35)	0.90–1.00 (35, 36)	2,300	794 (28)	43.7 (29)
Colonoscopy with polypectomy	0.95 (0.92, 0.99) (35)	0.90–1.00 (35, 36)	–	1,172 (28)	84–140

CMS: Centers for Medicare and Medicaid Services in the US; gFOBT: guaiac fecal occult blood test; FIT: fecal immunochemical test; mSEPT9: methylated SEPT DNA plasma assay; mt-sDNA: multi-target stool DNA test.

The face-to-face interviews were conducted with 5 experts at their workplaces or a private room where no one could observe or overhear the discussions. Online interviews were also conducted for 10 experts using Zoom, Tencent, or WeChat. The first researcher (W.M.W.) moderated the interviews using follow-up prompts for clarity, and requested for additional information when needed. The second researcher (S.S.T.) took field notes to assess gestures, facial expressions and other non-verbal communications of the participants, and provided technical support during the interviews. The interviews were audio-recorded using a smartphone. The interviews lasted for 40 min approximately, which ranged from 30 to 60 min. Following rigorous standardized approach for qualitative research, the data collection was terminated when data saturation was achieved.

After the interviews, a self-administered questionnaire was used to collect demographic characteristics of experts, including age, sex, educational level, occupation, professional title, duration of working in the area of CRC screening, and their roles in the area of CRC screening.

### 2.4 Data analysis

Audio recordings of the interviews were transcribed verbatim using iflyrec software. The transcripts were double-checked and transferred to MAXQDA 2020 software by the researchers. An iterative data collection and analysis were employed using a constant comparison approach to build concepts and categories according to the grounded theory approach, which provides a thorough procedure-oriented method for coding, including open, axial, and selective coding phases.

Open coding involves breaking down, analyzing, comparing, conceptualizing, and classifying data, through which the central concepts and categories were created. Axial coding further classified, condensed, and refined the categories to develop subcategories related to each main category. Selective coding, the third phase, further abstracted and summarized the main categories to form the core categories, and finally build a grounded theoretical model covering all the collected data (37). A theoretical saturation test was conducted during and after the process of coding until no additional issues or insights were identified (17). In this interview, no new concept or category was found when the transcripts of the 11th respondent were coded. Additional 4 experts were interviewed to confirm the theoretical saturation. To increase the reliability of our findings, two researchers coded all the transcripts independently. A third researcher checked the original data, coding, extracted concepts and categories, and compared and discussed any controversy to reach a consensus.

## 3 Results

More than 600 min of audio recordings were collected and transcribed verbatim, from which the concepts related to the aims of this study were extracted. And then 83 initial categories were derived from the concepts in the open coding phase. Then 37 subcategories and 10 main categories were summarized from the initial categories through axial coding. In the selective coding, four core categories were identified, including current modality for CRC screening (C1), factors influencing screening effectiveness (C2), optimization of CRC screening modality (C3), and prospects for development (C4) (Table 3).

**Table 3:** Categories extracted by open coding, axial coding and selective coding.

Four core categories	Ten main categories	Thirty-seven subcategories (no. of initial categories)	Eighty-three initial categories (no. of initial concepts)
**C1** Current modality for CRC screening	**M1** Screening programs ongoing in China	**S1** Program description (11)	Detailed protocol (15); scale of screening (6); year of screening (4); levels of the programs (4); adherence to initial test (15); adherence to colonoscopy (14); age at screening (10); fecal samples for FIT (14); wave of screening (6); questionnaire for RA (4); mobilization methods (9)
		**S2** Complicated situations (9)	Small sample in each selected province (4); planned sample size (5); higher participation rate in rural than in urban areas (3); low accuracy of questionnaire-based RA (12); one screening for multiple cancers (3); difficulty in participant recruitment (6); low adherence to RA/colonoscopy (12); use of various brand of FIT kits (4); multiple waves of screening (4)
	**M2** Adaptability to Chinese situation	**S3** Well-established methods (3)	Long-term utility in practice (5); high sensitivity (7); good performance in detection of early-stage lesions (4)
		**S4** Cheapness and convenience (4)	Non-invasiveness of initial test (1), simplicity (3), easy implementation (3), cheapness (5)
	**M3** Rooms for improvement	**S5** Disadvantages in general (3)	High false-positivity /low specificity (7); unstandardized procedure (5); poor quality control (5)
		**S6** Insufficient reliability of FIT results (5)	Inaccurate results (3); too many FIT kits available in market (3); inconsistent results by brands of kits (4); non-standardized unit (4); various cut-off values for positivity (5)
		**S7** Questionnaire to be improved (4)	Low accuracy (11); outdated risk factors (6); varied questionnaires across populations (7); poor pertinence of questions (11)
**C2** Factors influencing screening effectiveness	**M4** Multi-dimensional factors	**S8** Screening protocol (7)	Population adherence (13); scientific nature of screening (12); smoothness of process (10); personnel capacity (5); colonoscopy resources (7); convenience of method (8); degree of implementation (6)
		**S9** Propaganda and education (6)	Social impact (5); prevention priority (7); mobilization of primary care doctors (2); fear for colonoscopy examination (5); importance of secondary prevention (6); knowledge on colorectal cancer (5)
		**S10** Quality of CRC screening (5)	Adequate implementation (5); coordination (6); results interpretation (6); standardized personnel training (4); similar equipment (3)
		**S11** Implementation efforts of government (3)	Affordability (8); favored policy or medical insurance support for colonoscopy (6); green channel for diagnosis and treatment (4)
**C3** Optimization of CRC screening modality	**M5** Great potential of novel screening tests	**S12** Suitable for opportunistic screening (3)	Can be used for employee physical examination (8); can be used by medical centers (5); can be used as public health service (5)
		**S13** Recommended in guidelines (3)	Listed in guidelines (8); as alternative diagnostic methods (7); as intervention methods (4)
		**S14** Rapid development (3)	Decreasing cost (5); improvement in technologies (3); rapid upgrade (4)
	**M6** Advantages of novel screening tests	**S15** Can be uses as personalized screening tests (3)	Personalized test (5); precise screening (3); including genetic risks (2)
		**S16** More accurate than FIT (2)	More accurate (13); higher sensitivity (9)
		**S17** Provide more choices (7)	Decrease the risk of cross-infection (2); suitable for colonoscopy intolerant (9); avoid anesthesia accidents (2); suitable for detection of intestinal lesions (2); sensitive to inflammatory lesions (2); improve detection of adenoma (5); detect cancer metastasis (2)
		**S18** More convenient for subjects (3)	Can be implemented in route physical examination (4); simple and quick (8); no need for sick leave (7)
		**S19** Non-invasiveness nature (2)	Non-invasive (12); painless (12)	
**M7** Disadvantages of novel screening tests	**S20** Technology to be improved (8)	Localization of technologies (4); less accurate than reported (5); time consuming (8); various contraindications (9); unsuitable for colon screening (6); insufficient power driving colon capsule endoscopy (1); poor controllability (5); numerous factors influencing gut microbiota (2)		
	**S21** Effectiveness to be validated (3)	No evidence derived from large-scale screening (7); no evidence from Chinese populations (3); further validation (5)		
	**S22** Expensiveness (2)	Expensive test (13); high cost (14)		
	**S23** More requirement in technology (7)	Complex operation (10); high quality bio-specimen (2); high requirement for technicians (5); low accessibility (3); strict bowel preparation (12); inconvenience (2); high requirement for equipment (4)		
	**S24** Effectiveness inferior to colonoscopy (4)	No concomitant biopsy (4); less accurate results (7); need confirmatory colonoscopy (11); minimal lesions undetected (8)		
**M8** Optimization of modality for CRC screening	**S25** General suggestions (4)	Supplement with digital anal rectal examination (1); strengthen quality control (2); standardized procedure (2); lower age at screening (4)		
	**S26** Using quantitative FIT instead (8)	Quantitative results (2); much less products available in market (2), enable risk stratification (2); low false-positive rate (5); accurate fecal sampling (2); standardized test procedure (2); more accurate results (3); long storage time of samples (2)		
	**S27** Optimization of questionnaire (5)	Use electronic version (1); establish risk scoring (3); include genetic risk factors (1); multi-dimensional risk factors (5); update questionnaire if necessary (12)		
	**S28** Better colonoscopy service (4)	Better laxatives (1); more colonoscopy resources (9); use artificial intelligence (1); apply painless colonoscopy (5)
**C4** Prospects for development of CRC screening	**M9** Promising effects of CRC screening	**S29** Increasing disease burden of CRC (2)	Increasing disease burden (14); rapidly increasing incidence (12)
		**S30** Most cost-effective approach to control CRC (5)	Difficult primary prevention (10); cost-effective secondary prevention (9); removal of precancerous lesions (11); avoiding much higher treatment cost (14); preventive effect comparable to cervical cancer screening (4)
		**S31** New concepts on health promoted by screening (2)	To improve awareness on health (4); to pursue quality of life (2)
	**M10** Diverse development in CRC screening	**S32** Personalized screening (2)	Precise screening (2); including genetic risk factors (4)
		**S33** Screening procedure optimized further (3)	To refine implementation of screening (4); to optimize screening modality through multi-center cooperation (3); to increase coverage of screening (8)
		**S34** Higher adherence of screened populations (2)	To improve adherence to initial tests (13); to improve adherence to colonoscopy (12)
		**S35** More attention on specificity of screening tests (2)	To concern negative predictive value (4); to improve specificity (15)
		**S36** Better novel screening tests (2)	To lower screening cost (8); to improve accuracy (11)
		**S37** Strategies to enhance propaganda and education (3)	To eliminate fear for colonoscopy (12); to enhance propaganda and education on secondary prevention (8); to correct perceptions on screening (7)

CRC: colorectal cancer; FIT: fecal immunochemical test; RA: risk assessment.

### 3.1 Categories and sub-categories

#### 3.1.1 C1: current CRC screening modality in China

The national guideline of China for CRC screening recommended parallel use of RA and two-sample qualitative FIT as preliminary tests, followed by a colonoscopy follow-up (6). This triage screening strategy is being carried out as a major public health service in Shanghai (8), Guangzhou (9), and Hangzhou (11). In the CanSPUC program initiated in 2012 in mainland China, a risk scoring system was used as an initial test (12). In Taiwan and Hong Kong, one-sample quantitative FIT was used to identify high-risk individuals for subsequent colonoscopy (38, 39). Almost all experts expressed their concerns on the high false-positive rates of the preliminary tests, and proposed several potential contributors to the adverse situation.

First, various qualitative FIT products were used in screening programs as an initial test. Qualitative FIT is commonly used in China due to its convenience and cheapness. However, more than 10 brands of qualitative FIT kits produced by different manufacturers are available in China, but with low consistency in test results. For the quantitative FIT, on the contrary, only one product with a brand of OC-MICRO is offered dominantly in China.

“Quantitative FIT is widely used in developed countries or areas, while qualitative FIT is more commonly-used in China. The low consistency of the results tested by different brands of qualitative FIT kits have become a big problem in the practice of CRC screening.” (Male, researcher, ID14)

“The results of qualitative tests are not consistent for FIT kits produced by different manufacturers. However, different FIT kits were used across screening programs; even in a CRC screening program, the FIT kits may change year by year.” (Female, CDC staff, ID7)

Second, the recommended RA tools have not been updated. The risk factors of CRC may have changed along with the social development and nutritional transition in China. However, the RA tools have not been updated based on newly-established risk predictive models or risk scoring systems.

“The questionnaire currently used was derived from several case-control studies conducted in Jiashan County, Zhejiang Province, several decades ago, and was just simplified in a large-scale national cancer screening program in recent years. Obviously, the questionnaire is outdated. We have suggested to update the questionnaire during the past years.” (Male, primary care provider, ID1)

“Generally, people were less likely to attend follow-up colonoscopy if they were identified as high-risk individuals by questionnaire-based RA only. The risk stratification based on the results of the questionnaire was not convincing for our subjects.” (Female, primary care provider, ID9)

Finally, various misconducts may happen in each part of the whole procedure of screening, which may have led to inaccurate test results.

“Most subjects would report having constipation or diarrhea even if they had the symptoms occasionally, if doctors did not explain related definitions very clearly during the survey. This would lead to low quality of the collected data and incorrect risk stratifications” (Male, gastroenterologist, ID2)

“The participants were asked to collect fecal samples by themselves. However, it is difficult for them to collect appropriate amount of stool samples. Some participants even added water into the tubes …. The operation was definitely unstandardized and incorrect.” (Female, researcher, ID12)

#### 3.1.2 C2: factors influencing screening effectiveness

The experts’ opinions on barriers and facilitators for effectiveness of CRC screening were also collected. Specifically, the experts were highly concerned about the population adherence, rationality, and affordability of CRC screening tests. As presented in Table 4, the accuracy and the affordability ranked the first and the second of the four important characteristics of screening tests (accuracy, affordability, accessibility and non-invasiveness) according to the viewpoints of the experts.

**Table 4:** The concerns of experts on the characteristics of screening tests for colorectal cancer.

	Rank of concerns on screening tests				Total score
	First	Second	Third	Fourth	
Accuracy	11	0	3	1	51
Affordability	0	8	4	3	35
Accessibility	3	2	5	5	33
Non-invasiveness	1	5	3	6	31

The concerns ranking first, second, third or fourth assigned with 4, 3, 2 or 1 score, respectively.

**Figure 2:**
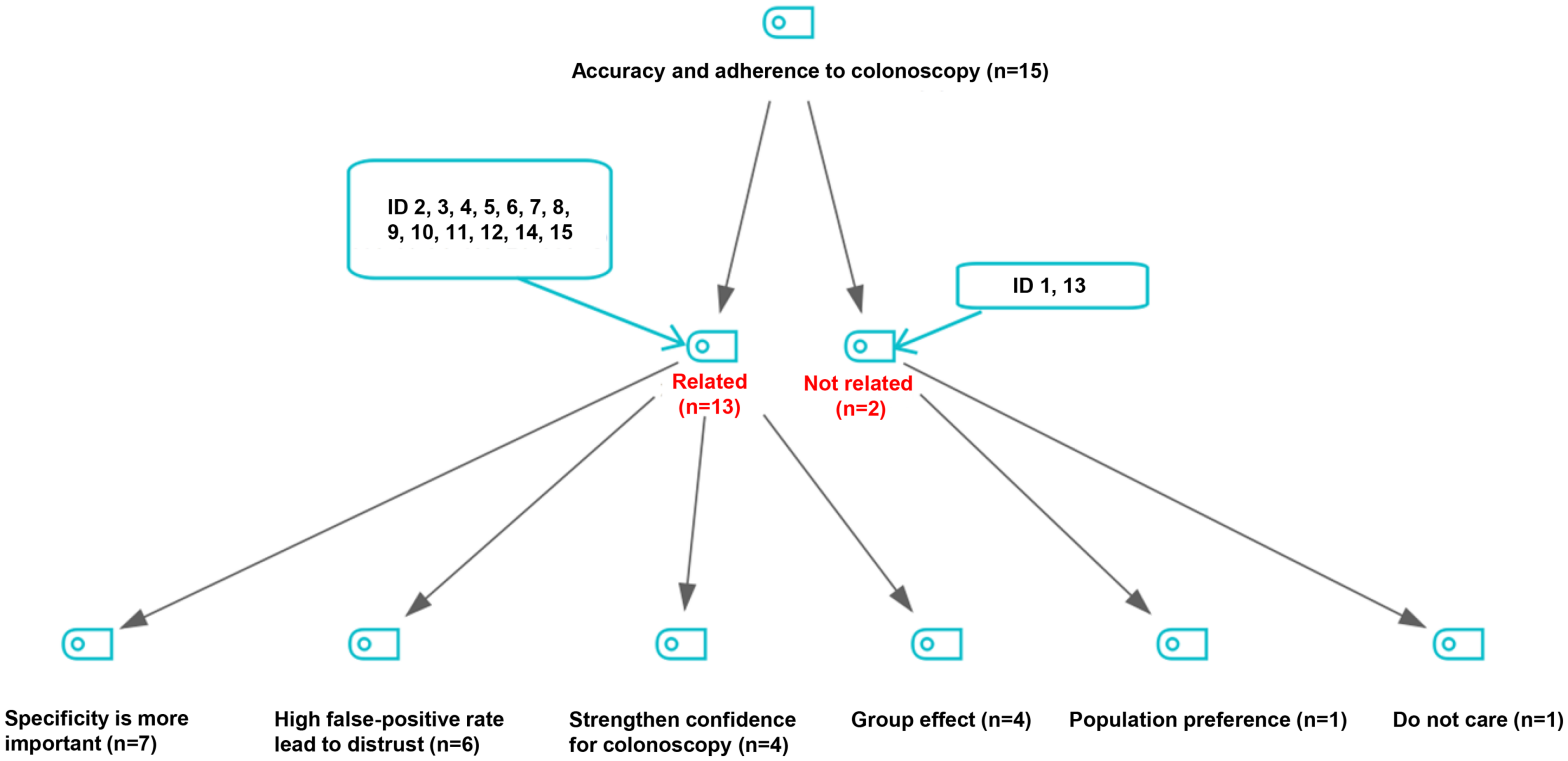
Viewpoints on the relationship of accuracy of initial screening tests with adherence to follow-up colonoscopy.

As shown in Figure 2, a total of 13 experts believed that adherence to subsequent colonoscopy was related to the accuracy of FIT. Among them, 7 experts proposed that the specificity of FIT was more important than other indices of accuracy, 6 experts thought that high false-positive rate of FIT may have led to the distrust of screening results in screened populations, 4 experts believed that higher accuracy of initial test results strengthen confidence for attending the subsequent colonoscopy examination, and 4 experts mentioned the possible group effect in attending subsequent colonoscopy.

“The CRC screening was carried out round by round, and multiple waves of screening have been conducted in many places. Unfortunately, a vicious circle was observed due to high false-positive rate of the initial screening test: a high false-positive rate of an initial test may decrease the adherence to subsequent colonoscopy, and further impedes the sustainability of CRC screening programs.” (Female, researcher, ID6)

“For example, a subject identified as high-risk by initial screening tests may feel tricked, if he/she was not found any lesions in subsequent colonoscopy examination. Then he/she would express his/her distrust of the initial test results to his/her peers, which may further decrease adherence to colonoscopy in the community” (Female, CDC staff, ID11)

#### 3.1.3 C3: optimization of CRC screening modality

Despite the problems existing in current screening modality, the experts believed that the modality could be optimized through improving process management (S25), applying quantitative FIT (S26), updating RA tools (S27), and providing better colonoscopy service (S28) (Table 3). Particularly, the experts suggested to enhance the pertinence of factors for RA by excluding outdated questions like chronic appendicitis or appendectomy, chronic cholecystitis or cholecystectomy, chronic constipation and chronic diarrhea, and adding several important risk factors into the system, such as body mass index (BMI), physical activity, aspirin use, diet and smoking.

“We now use the questionnaire-based RA derived from the surveys in Jiashan County and Haining City of Zhejiang Province in the 1990s. The RA tool may be outdated, and did not exactly reflect the risk exposures nowadays. The risk factors of CRC have been changing over time, and BMI, physical activities and smoking should be included in the questionnaire now.” (Female, CDC staff, ID7)

The experts also proposed to use 1-sample FIT instead of 2-sample tests, or apply quantitative FIT instead of inaccurate qualitative tests. Regarding the affordability, most experts thought that the cost of 20 CNY per quantitative test is acceptable in China.

“For the FIT, I recommend 1-sample FIT instead of 2-sample tests according to the results of our screening program, and as most countries did.” (Male, researcher, ID14)

“Recently, I heard that an agency of OC-MICRO (a brand of FIT) in Hangzhou provided the cost per capita of 20 CNY. The government of Zhejiang Province has launched a program for early detection of cancer, and intends to use quantitative FIT, but I don't know whether they are using it this year.” (Male, primary care provider, ID1)

“In my opinion, there is no problem with CRC screening tests and the key is to fully realize the effectiveness of each part in the whole screening process. However, a big gap existed between the observed and the expected effectiveness of a certain screening program. I think the most important is to fully realize the expected effectiveness of each test in CRC screening practice.” (Female, researcher, ID12)

#### 3.1.4 C4: prospects for development of CRC screening

The much higher costs of the novel screening tests for CRC than RA, FIT and even colonoscopy would limit the widespread use of these novel tests in China, a country with a huge population and insufficient medical resources. As shown in Table 2, the cost of colonoscopy is far lower in China (44 USD) than in the United States (2,300 USD), which devalue and restrict the application of the available novel CRC screening tests in China.

In this study, nine experts (ID 1, 4, 5, 7, 8, 9, 12, 13, 14) believed that none of the available novel CRC screening tests is good enough to replace the triage screening methods that are currently used. However, they acknowledged that several novel tests can be used as an alternative screening method, and have great potential in opportunistic screening. Five experts (ID 2, 3, 10, 11, 15) believed that mt-sDNA test is the most promising novel method for CRC screening in the near future.

“Due to the relatively high cost, the novel tests may be used as the alternative methods in personalized service. However, the government can merely provide basic public health services but not the personalized ones. We can provide the residents with different alternatives, but it is not economically practical for government to cover all the expenses.” (Male, health official, ID5)

“The mt-sDNA test can help to improve sensitivity and increase participation rate of screening … you can adjust threshold of mt-sDNA test for more targeted screening, which I think is a good thing. The mt-sDNA test includes quantitative molecular assays for hemoglobin, genetic mutations, methylation, etc. I think it is of great significance for CRC screening.” (Male, gastroenterologist, ID3)

### 3.2 Model construction

**Figure 3:**
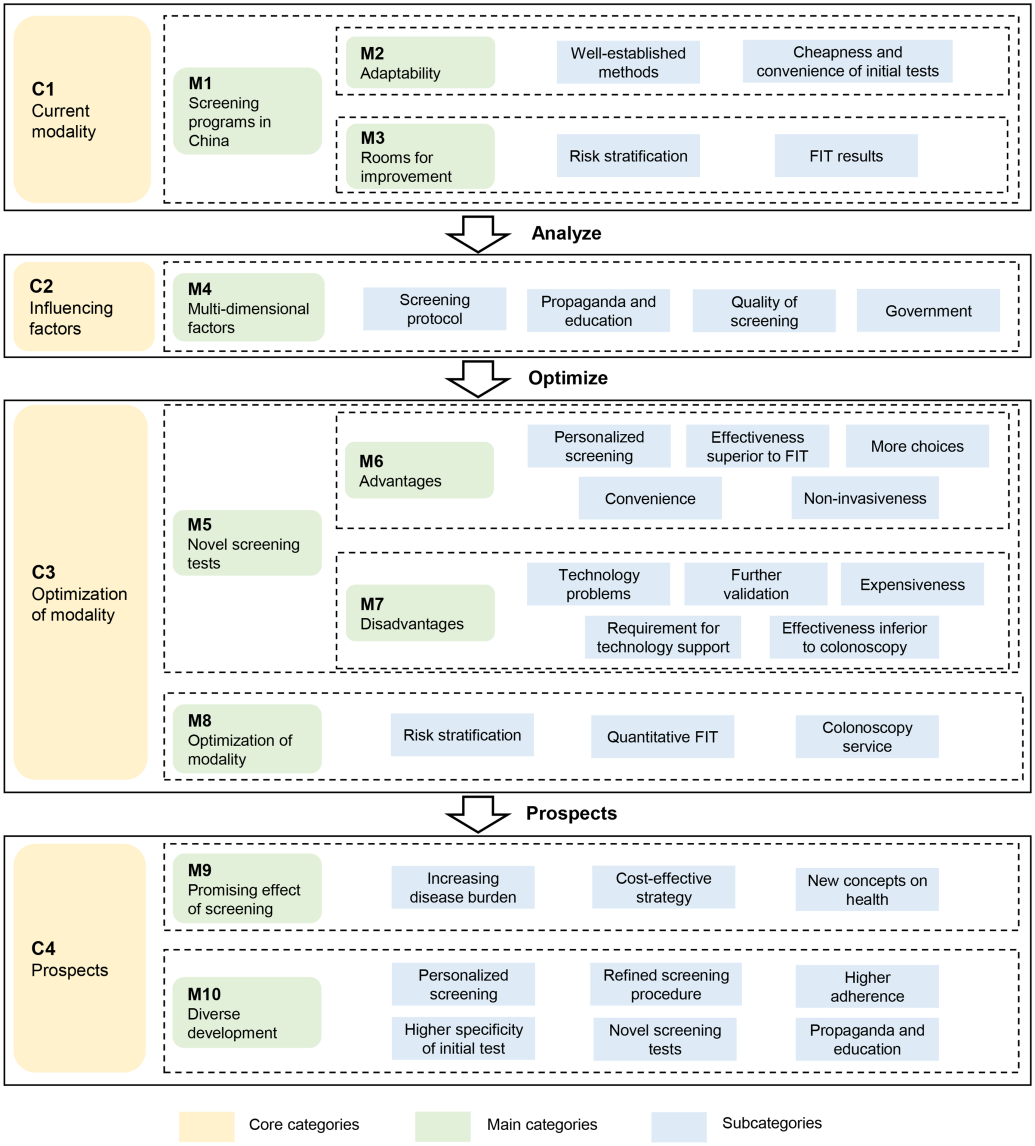
The grounded theory-based framework to evaluate the screening strategies for colorectal cancer. FIT: fecal immunochemical test.

Based on the subcategories, main categories and core categories identified in Table 3, the essential issues in CRC screening in China can be summarized into a grounded theoretical model in Figure 3. Over the past decades, the triage screening modality utilized in CRC screening programs (M1) has been found quite suitable for China and widely adopted in the country due to its cheapness and convenience (M2). However, there are several challenges (M3), particularly the suboptimal adherence to colonoscopy that has greatly lowered the effectiveness of screening programs. Factors regarding health propaganda and education, quality control and government might also influence the effectiveness of screening programs (M4). Fortunately, the CRC screening programs can be improved by applying novel screening tests (M5) or optimizing current screening modality (M8). Despite the advantages and good prospects of the novel screening tests (M6), their high costs and high technical requirement have restricted their application in large-scale screening programs (M7). Therefore, optimization of current screening modality (M8) is highly anticipated, which can be achieved by using more accurate risk stratification, applying quantitative FIT, and providing better colonoscopy service. In the opinions of the experts interviewed, considering the generally acknowledged effectiveness of CRC screening (M9), the diverse development in CRC screening is highly expected, including personalized screening, optimized screening procedure, higher population adherence, higher specificity of initial tests, use of novel screening tests, and improved health education (M10).

## 4 Discussion

In this study, we explored the viewpoints of 15 experts regarding the CRC screening modality in China through semi-structured interviews. Generally, the experts responded positively to the triage screening modality adopted in China, but also expressed concerns on the low adherence to colonoscopy among high-risk subjects who were identified by initial screening tests. The experts suggested to optimize the initial screening test by using quantitative FIT instead of the qualitative one, and by updating the RA tools currently-used in China. For the emerging screening technologies, the experts proposed to use the novel tests as supplementary methods to the triage screening modalities. The opinions may help to optimize the currently-used screening modality for CRC, improve adherence to follow-up colonoscopy, and fully achieve the effectiveness of screening programs in China.

The effectiveness of CRC screening programs depends on the accuracy of screening tests and the adherence of screened populations (40). A low adherence to colonoscopy was consistently observed among high-risk subjects identified by initial tests in China (8, 12). In this study, most experts believed that adherence to colonoscopy was influenced by the accuracy of FIT, the most commonly used initial test in triage screening for CRC globally. In our previous studies, we found that adherence to colonoscopy was positively associated with specificity and positive predictive value of initial screening tests (14, 15), which was in line with the findings in other populations (41, 42). Therefore, the high false-positive rate of initial tests in China was the common concern of the experts in this study.

To release the concern, the experts proposed two approaches to decrease false-positive rate of the initial tests, which may help to improve the adherence to subsequent colonoscopy in China. First, the expert recommended to use quantitative FIT instead of the qualitative one. The quantitative FITs outperform the qualitative ones not only due to its higher accuracy, but also for its flexible cut-off values (43). However, quantitative FIT remains to be developed for the large-scale screening practices in China due to its relatively higher cost. Moreover, the sensitivity and specificity of one-sample qualitative FIT was found similar to those of multiple-sample tests, regardless of the brand of FIT products (27). Evidently, one-sample FIT should be adopted in China to simplify the screening procedure, improve participation rate, and reduce demands for colonoscopy (44). Second, the experts proposed to update the RA tools. The currently-used RA tool did not include age, sex, smoking, drinking, BMI, diet, physical activity, diagnosis of diabetes, use of non-steroidal anti-inflammatory drugs or aspirin (8, 11, 12), the common risk factors of CRC included in other risk scoring systems (45, 46) and recommended in the updated Chinese guideline for CRC screening (32). It was also found that several factors for RA can be removed from questionnaire without any additional missed CRC cases (47). Thus, the RA tools is urgently needed to be updated using population-and period-specific risk factors of CRC for better accuracy and applicability.

In recent years, many novel CRC screening tests have been developed to replace or reduce invasive examination like colonoscopy. However, the inconsistent performance of these tests in populations has limited their widespread applications (32, 48). After all, the accuracy is the most important feature of a screening test. In this study, we found that the experts ranked affordability the second most important feature of screening tests, particularly for large-scale screening programs. Most novel screening tests are expensive and sometimes require additional technical support. For example, about 512 USD is needed for one mt-sDNA test, much lower than 1,172 USD for a colonoscopy in the US. Therefore, mt-sDNA test is recommended in the guideline probably for the consideration of cost-effectiveness. In China, however, only 44 USD is needed for a colonoscopy examination, much lower than the costs in western countries (28, 29). Therefore, it is more cost-effective to apply colonoscopy in China, the reference test in CRC screening. So far, the novel tests are suggested to be used in opportunistic screening in physical examination institutions or hospitals.

This study has several limitations. First, we did not interview the participants of CRC screening programs, who may provide additional opinions on the CRC screening programs in China. However, as this qualitative interview study was designed from the perspective of service providers, and the opinions of the experts were derived from their experience and interactions with the screened subjects, our results have great values for evidence-based policy making. Second, the interview was conducted in the Chinese setting with country-specific policy, realistic condition, and academic issues, which inevitably brings the question of whether the model was universal and applicable in other countries. Finally, the conclusions of this study were made through a theoretical discussion, not based on a real-world screening data analysis. However, we summarized the sensitivity, specificity and price of each currently-used initial screening tests through comprehensive literature reviews, and provided the information to our subjects at the interview, which may have made the discussion evidence-based.

## 5 Conclusion

In the opinions of experts in China, the triage screening modality, if improved, remains the optimal choice for Chinese populations. To use quantitative FIT or update RA tools may help to identify high-risk individuals more accurately, improve adherence to subsequent colonoscopy, and thus fully achieve the effectiveness of screening. The emerging novel technologies have great potentials in opportunistic CRC screening in China as supplementary tests. Further studies are needed to verify and improve the grounded theoretical model developed in this study, and apply the theoretical results into the real-world screening practices.

## Data availability statement

The raw data supporting the conclusions of this article will be made available by the authors, without undue reservation.

## Author contributions

WX conceived and designed the study. WW, YC, and MCW made substantial contributions to the study design. WW drafted the manuscript. WW and ST contributed to data collection and data sorting. ST and JH contributed to data analysis. All authors contributed to the article and approved the submitted version.
